# Melatonin's role in managing nocturia in Parkinson disease: A critical examination of recent studies

**DOI:** 10.1111/ene.16220

**Published:** 2024-01-18

**Authors:** Yu‐Hsiang Lin, Horng‐Heng Juang

**Affiliations:** ^1^ Department of Urology Chang Gung Memorial Hospital at Linkou Taoyuan Taiwan; ^2^ School of Medicine Chang Gung University Taoyuan Taiwan; ^3^ Department of Anatomy, School of Medicine Chang Gung University Taoyuan Taiwan

Dear Editor,

We were deeply intrigued by the study conducted by Batla et al. [1] on the use of melatonin in nocturia among Parkinson disease patients. Their research presents a compelling case, demonstrating the efficacy of a 2‐mg dose of melatonin in significantly reducing nighttime urine volume and the number of voiding episodes during the night. This finding is particularly relevant to the issue of nocturia in men with prostate enlargement. In urology, the impaired bladder capacity that results from prostate enlargement, and subsequently contributes to nocturia, is typically managed with alpha‐blockers. However, when patients present with nocturnal polyuria, the approach shifts to using antidiuretic hormone supplements like desmopressin.

In a groundbreaking correlation, Obayashi et al. [2] found a significant negative relationship between nocturia and urinary 6‐sulfatoxymelatonin levels, a metabolite of melatonin. This suggests that melatonin supplementation might effectively reduce nocturia through its influence on antidiuretic hormone action. Despite concerns about the risk of hyponatremia associated with antidiuretic hormones, Batla's study offers promising prospects. Melatonin, particularly in its endogenous form, appears to be a safer and more effective alternative to exogenous desmopressin, especially because endogenous antidiuretic hormones are less likely to produce hyponatremia.

Furthermore, studies have revealed a dosage‐dependent effect of melatonin on antidiuretic hormone (ADH) secretion. Animal studies consistently show that melatonin inhibits ADH secretion [3]. In human subjects, a higher dose of melatonin, approximately 5 mg, tends to suppress ADH secretion, whereas a lower dose of 0.5 mg appears to stimulate it [4]. This suggests a nuanced interaction between melatonin dosage and ADH activity. Conversely, Drake et al.'s [5] findings indicate that a 2‐mg dose of melatonin, despite not significantly altering nighttime urine volume or frequency, effectively reduced the distress associated with nocturia.

The contrasting results between Batla's study, where a 2‐mg dose of melatonin was effective, and Drake's study, where a 2‐mg dose showed limited effects, illuminate the complexity of melatonin's impact on nocturnal polyuria. These discrepancies underscore the challenges faced in standardizing a melatonin dosage for nocturnal polyuria treatment. Given the variable individual responses to melatonin, influenced by factors such as patient weight, which can affect the blood concentration of the drug, prescribing a one‐size‐fits‐all dosage becomes problematic.

It becomes evident that an upstream approach, akin to the regulatory mechanisms seen with testosterone, follicle‐stimulating hormone, and luteinizing hormone in the hypothalamic–pituitary–gonadal axis, might be more effective in formulating treatment guidelines for nocturnal polyuria. This perspective not only addresses the dosage variability but also underscores the intricate balance required in hormonal regulation for optimal therapeutic outcomes.

## CONFLICT OF INTEREST STATEMENT

The authors have no conflicts of interest to declare.

## Data Availability

Research data are not shared.
